# For Whom Does Determinism Undermine Moral Responsibility? Surveying the Conditions for Free Will Across Cultures

**DOI:** 10.3389/fpsyg.2019.02428

**Published:** 2019-11-05

**Authors:** Ivar R. Hannikainen, Edouard Machery, David Rose, Stephen Stich, Christopher Y. Olivola, Paulo Sousa, Florian Cova, Emma E. Buchtel, Mario Alai, Adriano Angelucci, Renatas Berniûnas, Amita Chatterjee, Hyundeuk Cheon, In-Rae Cho, Daniel Cohnitz, Vilius Dranseika, Ángeles Eraña Lagos, Laleh Ghadakpour, Maurice Grinberg, Takaaki Hashimoto, Amir Horowitz, Evgeniya Hristova, Yasmina Jraissati, Veselina Kadreva, Kaori Karasawa, Hackjin Kim, Yeonjeong Kim, Minwoo Lee, Carlos Mauro, Masaharu Mizumoto, Sebastiano Moruzzi, Jorge Ornelas, Barbara Osimani, Carlos Romero, Alejandro Rosas López, Massimo Sangoi, Andrea Sereni, Sarah Songhorian, Noel Struchiner, Vera Tripodi, Naoki Usui, Alejandro Vázquez del Mercado, Hrag A. Vosgerichian, Xueyi Zhang, Jing Zhu

**Affiliations:** ^1^Department of Law, Pontifical Catholic University of Rio de Janeiro, Rio de Janeiro, Brazil; ^2^Department of History and Philosophy of Science, University of Pittsburgh, Pittsburgh, PA, United States; ^3^Department of Philosophy, Florida State University, Tallahassee, FL, United States; ^4^Department of Philosophy, Rutgers University, New Brunswick, NJ, United States; ^5^Tepper School of Business, Carnegie Mellon University, Pittsburgh, PA, United States; ^6^Institute of Cognition and Culture, Queen’s University, Belfast, United Kingdom; ^7^Department of Philosophy, University of Geneva, Geneva, Switzerland; ^8^Department of Psychology, The Education University of Hong Kong, Tai Po, Hong Kong; ^9^Department of Pure and Applied Sciences, University of Urbino Carlo Bo, Urbino, Italy; ^10^Institute of Psychology, Vilnius University, Vilnius, Lithuania; ^11^School of Cognitive Science, Jadavpur University, Kolkata, India; ^12^Department of Philosophy, Seoul National University, Seoul, South Korea; ^13^Department of Philosophy and Religious Studies, Utrecht University, Utrecht, Netherlands; ^14^Institute of Philosophy, Vilnius University, Vilnius, Lithuania; ^15^Instituto de Investigaciones Filosóficas-UNAM, Mexico City, Mexico; ^16^Independent Researcher, Tehran, Iran; ^17^Department of Cognitive Science and Psychology, New Bulgarian University, Sofia, Bulgaria; ^18^Department of Social Psychology, University of Tokyo, Tokyo, Japan; ^19^Department of History, Philosophy and Judaic Studies, Open University of Israel, Ra’anana, Israel; ^20^Department of Philosophy, American University of Beirut, Beirut, Lebanon; ^21^Department of Psychology, Korea University, Seoul, South Korea; ^22^Sloan School of Management, Massachusetts Institute of Technology, Cambridge, MA, United States; ^23^CLOO Behavioral Insights Unit, Porto, Portugal; ^24^School of Knowledge Science, Japan Advanced Institute of Science and Technology, Ishikawa, Japan; ^25^Department of Philosophy and Communication Studies, University of Bologna, Bologna, Italy; ^26^Faculty of Social Sciences and Humanities, Universidad Autónoma de San Luis Potosí, San Luis Potosí, Mexico; ^27^Munich Center for Mathematical Philosophy, Ludwig Maximilians Universität, Munich, Germany; ^28^Department of Philosophy, National University of Colombia, Bogotá, Colombia; ^29^Faculty of Philosophy, Scuola Universitaria Superiore IUSS, Pavia, Italy; ^30^Faculty of Philosophy, Vita-Salute San Raffaele University, Milan, Italy; ^31^Department of Philosophy and Educational Sciences, University of Turin, Turin, Italy; ^32^Department of Humanities, Mie University, Tsu, Japan; ^33^School of Humanities, Southeast University, Nanjing, China; ^34^School of Information Management, Sun Yat-sen University, Guangzhou, China

**Keywords:** free will, compatibilism, cognitive style, situationism, dispositionism, sourcehood, alternate possibilities

## Abstract

Philosophers have long debated whether, if determinism is true, we should hold people morally responsible for their actions since in a deterministic universe, people are arguably not the ultimate source of their actions nor could they have done otherwise if initial conditions and the laws of nature are held fixed. To reveal how non-philosophers ordinarily reason about the conditions for free will, we conducted a cross-cultural and cross-linguistic survey (*N* = 5,268) spanning twenty countries and sixteen languages. Overall, participants tended to ascribe moral responsibility whether the perpetrator lacked sourcehood or alternate possibilities. However, for American, European, and Middle Eastern participants, being the ultimate source of one’s actions promoted perceptions of free will and control as well as ascriptions of blame and punishment. By contrast, being the source of one’s actions was not particularly salient to Asian participants. Finally, across cultures, participants exhibiting greater cognitive reflection were more likely to view free will as incompatible with causal determinism. We discuss these findings in light of documented cultural differences in the tendency toward dispositional versus situational attributions.

## Introduction

The question of whether free will is compatible with determinism has fueled an intricate debate among philosophers (Strawson, 1963; Frankfurt, 1969; Fischer et al., 2009), which recent scientific work on human volition has enlivened (Dennett, 2004; Mele, 2008; Caruso, 2012). The crux of the free will problem can be outlined succinctly: In a deterministic universe, all events are the consequence of past events and the laws of nature. Then, if our universe is deterministic and every human action is the consequence of past events and the laws of nature, do people exercise control over their behavior in the way required for moral responsibility? Philosophical debate on this question has clustered around two proposed conditions for free will: *alternate possibilities* and *ultimate sourcehood*.

According to the *principle of alternate possibilities*, free will and moral responsibility depend upon the ability to do otherwise. Since determinism implies that agents could not have done otherwise once initial conditions and the laws of nature are held fixed, it follows that free will and moral responsibility are incompatible with determinism. This conclusion was challenged when philosopher Harry Frankfurt (1969) – in an ingenious appeal to counterfactual intervention *–* provided an influential argument against the principle of alternate possibilities, illustrated in the following thought experiment: Suppose that I want to stay home all day on Sunday to rest for the busy week ahead. Come Sunday, I cancel my plans to go hiking with friends. Instead, I spend the day watching a movie, cooking a meal, and taking a long nap on the couch. Unbeknownst to me, the door to my apartment was jammed and I would not have been able to go hiking, or leave at all, had I tried. In this circumstance I could not have done otherwise; but did I *freely* stay home anyway? According to the principle of alternate possibilities, I did not; but Frankfurt had the influential insight that we should think of my behavior as being freely willed despite my lack of alternate possibilities. Do people ordinarily conceive of free will as Frankfurt does? Some evidence has shown that North Americans typically agree with Frankfurt’s assessment that alternate possibilities are unnecessary for free will or moral responsibility (Miller and Feltz, 2011).

Determinism doesn’t merely entail the absence of alternate possibilities. In a deterministic universe, the laws of nature together with a complete description of the state of the universe at time *t*_1_ entail the state of the universe at time *t*_2_. Thus, agents never initiate the causal sequence resulting in their behavior; rather, people’s intentions, desires, and reasons are themselves the result of prior events. Hence, determinism also precludes what is known as *ultimate sourcehood* of one’s own acts (Pereboom, 2001): For example, if in a deterministic universe I make a salad instead of a burger for lunch, the causal chain of events that can be traced back from my action does not stop at my intentions, desires, and beliefs, but regresses to events that predate my own existence.

The same is true of people’s achievements and crimes: In a deterministic universe, agents may proximately cause them, but their ultimate source traces back to the beginning of time. Some *incompatibilist* philosophers view this lack of sourcehood as undermining free will and moral responsibility (van Inwagen, 1983; Pereboom, 2001), while some *compatibilist* philosophers do not. In turn, non-philosophers have been shown to endorse incompatibilism when given an abstract description of a deterministic universe (Nichols and Knobe, 2007), and to endorse compatibilism when given a concrete description of a deterministic universe (Nahmias et al., 2005).

Thus, existing empirical evidence reveals that laypeople hold agents morally responsible, even while acknowledging that the agent could not have done otherwise (Miller and Feltz, 2011), and did not originate the causal sequence resulting in action (Nichols and Knobe, 2007). However, past studies share an important limitation: With one notable exception (Sarkissian et al., 2010), they have been administered only to relatively small and homogeneous samples of respondents in the United States. Since cultures differ considerably in their values (Hofstede et al., 2010) and philosophical judgments (Machery et al., 2004; Machery, 2017), whether past findings generalize across cultures remains very much an open question. Furthermore, given growing concerns about the replicability of key results in experimental psychology and philosophy (Cova et al., 2018), conducting a highly powered, cross-cultural generalization of core findings is a timely and important enterprise. Thus, our primary goal in this paper is to draw on a large and diverse sample of international respondents in order to examine intuitions regarding the conditions for free will and moral responsibility across cultures. In addition, the present study offers two novel contributions.

First, motivated by evidence of cultural variation in explanatory style, we examine whether Asians and Westerners differ in their reasoning about free will and moral responsibility. Numerous experiments on social attribution have shown that people typically allude to an agent’s *internal* characteristics, such as her skills, desires, or personality, when attempting to explain why and how she behaves (Heider, 1958; Weiner, 1985) – a tendency known as *dispositionism*. For instance, when asked to describe a medicine mix-up at a children’s hospital, many participants say the accident happened because a particular pharmacy worker was “careless” or “irresponsible” (Chiu et al., 2000). Yet, a handful of comparative studies have provided compelling evidence that dispositionism may not be universally shared (Miller, 1984; Morris and Peng, 1994; Choi et al., 1999): In particular, East Asians are more likely to highlight features of the situation. For instance, when asked to describe the same hospital incident, Chinese participants tended to attribute this occurrence to extrinsic causes: e.g., the clinic’s failure to “assure the quality of the medicine” or “monitor the performance of the workers.” These differences in attributional style have been observed in naturalistic contexts too (Morris and Peng, 1994): When explaining a gruesome murder, an American newspaper (*The New York Times*) focused more on intrinsic features, while a Chinese newspaper (*World Journal*) focused more on situational factors.

Developmental evidence on the emergence of free will beliefs has revealed corresponding cultural differences: Between ages four and eleven, children in the United States appear to strengthen their belief that individuals can choose to violate norms (e.g., to make a classmate cry), whereas Nepali children show no such change (Chernyak et al., 2013). In comparison to the characteristically Western sense that agents choose whether to act on desires and social norms, Asian cultures are more likely to treat such situational factors as direct constraints on human behavior. Taken together, these findings suggest that intuitions regarding the conditions for free will may be different in Asian cultures.

Our second novel contribution is to characterize the motivational and cognitive underpinnings of compatibilist versus incompatibilist intuitions. Previous empirical research has shown that, at least among Americans, extraverts are more likely to view free will as compatible with determinism, while introverts are more likely to see them as incompatible (Feltz and Cokely, 2009) – a pattern that holds even among philosophers with expert knowledge about this very issue (Schulz et al., 2011).

Thus far, no research has sought to clarify why extraverts and introverts might differ by probing into their divergent reasoning styles – and our present study helps to fill this gap: Leading theories of extraversion point not only to greater interpersonal engagement, but also to differences in decision-making style (Depue and Collins, 1999). Specifically, introverts tend to adopt a rational thinking style (e.g., “I enjoy intellectual challenges”), while extraversion is more associated with an experiential thinking style (e.g., “I like to rely on my intuitive impressions”) and a dependence on habitual over goal-directed learning mechanisms (Pacini and Epstein, 1999; Skatova et al., 2013). A related literature suggests that extraverts are more reward-sensitive (Smillie, 2013), as evidenced by neuroimaging studies of gambling behavior (Cohen et al., 2005).

These contrasting styles of information and reward processing point to a potential explanation for the link between personality and compatibilist judgments: While introverts may tend to reflect more on the implications of determinism for free will, extraverts may be more immediately motivated to punish the perpetrator – consistent, also, with evidence that exacting third-party punishment is rewarding (de Quervain et al., 2004). To investigate this hypothesis, participants were asked to complete the Cognitive Reflection Test – a performance-based measure of individuals’ tendency to reconsider their initial intuitions (Frederick, 2005).

In the sections that follow, we report the methods and results of our large-scale, cross-cultural study. We end with a general discussion that connects our findings to our two novel contributions, and which emphasizes the importance of our findings, and cross-cultural work more generally, for philosophical methodology.

## Materials and Methods

### Participants

We invited 5,268 participants (46% women; age: *Median* = 26, *Min* = 18, *Max* = 88) from 21 different locations around the world to complete a survey as part of an international collaboration. Supplementary Table S1 provides details regarding the demographic profile, language, and medium of administration for each location.

### Procedure

As part of a battery of questions concerning philosophical thought experiments, participants were randomly assigned to read one of two hypothetical murder scenarios narrated in the primary local language. For each site, we sought a bilingual translator to prepare all stimuli in the local language, except where previously validated and published translations were already available (as noted in the section “Measures”).

Half of our participants (*n* = 2,381) read the “Actual Sequence” (AS) scenario, previously employed in influential studies of moral psychology (Nichols and Knobe, 2007; Feltz et al., 2009; Cova et al., 2012; Rose and Nichols, 2013; Feltz and Cova, 2014; Murray and Nahmias, 2014).^1^ The AS scenario opens by situating the reader in a deterministic universe:

Imagine a universe in which everything that happens is completely brought about by whatever happened before it. This is true from the very beginning of the universe, so what happened in the beginning of the universe brings about what happened next, and so on right up until the present. For example, 1 day John decided to have vegetable soup at lunch. Like everything else, this decision was completely brought about by what happened before it. So, if everything in this universe was exactly the same up until John made his decision, then it had to happen that John would decide to have vegetable soup at lunch.

Thus, the first paragraph describes a deterministic universe in which agents (a) could not have done otherwise, and (b) are not the source of their actions. Then, the second paragraph describes a hypothetical agent carrying out a murder in those precise conditions:

In this universe, a man named Bill has become attracted to his secretary, and he decides that the only way to be with her is to kill his wife and three children. Before he leaves on a business trip, he sets up a bomb that destroys his house and kills his family while he is away.

A separate group of participants (*n* = 2,887) read a variant of the “Counterfactual Intervener” (CI) case (adapted from Frankfurt, 1969; Miller and Feltz, 2011) – in which the perpetrator is the source of the murder, but happens to lack alternate possibilities:

The year is 3072. A group of mad scientists has invented a sophisticated device that can monitor what is going on in a person’s mind. The device works at a distance by sending and receiving signals from a special chip that can be easily implanted into a person’s brain. With the device, the scientists can change a person’s decisions to engage in specific actions by simply sending signals to the special chip implanted in the person’s head and thereby manipulating the activation of the person’s neurons.
One day, the scientists had a person infiltrate a clinic to find people so that the chip could be secretly implanted in them. Martin is one of the subjects who receive the implant.
The next day, while monitoring Martin’s thoughts, the scientists see that Martin is deliberating on a matter of great concern: whether to kill his friend Adam, who is having an affair with Martin’s wife. The scientists agree that they will let Martin make his own decision, but that, if he decides not to kill Adam, they will make him change his mind by sending signals that reinforce his desire and reasons to kill Adam. In other words, regardless of Martin’s own final decision, Martin will kill Adam, because the scientists are set on interfering if necessary. Martin decides to kill Adam and ends up killing Adam. The scientists didn’t have to interfere.

### Measures

This study made use of four dependent measures, presented consecutively. Two measures assessed non-moral aspects of free will, namely:

(1) *Freedom*: i.e., whether or not the agent acted freely when he killed his victim (1: yes, 0: no), and
(2) *Control*: i.e., how much control he had over killing his victim (1: no control – 7: complete control).

In two further questions, we examined whether participants judged that the agent should be held morally responsible:

(3) *Blame*: i.e., to what extent he was blameworthy (1: not at all – 7: extremely), and
(4) *Punishment*: i.e., how much punishment he deserved (1: no punishment – 7: severe punishment).

At the end of the survey, participants completed two further measures: the original (three-item) Cognitive Reflection Test (CRT; Frederick, 2005) and the Ten Item Personality Inventory (TIPI; Gosling et al., 2003). The CRT is a short assessment of participants’ cognitive style through three open-ended questions. For instance, one question asks: “*If it takes 5 machines 5 min to make 5 widgets, how long would it take 100 machines to make 100 widgets?”* Many participants provide the incorrect, intuitive answer that springs to mind, i.e., “100 min,” while others provide the correct answer, “5 min.” As an individual difference measure of cognitive style, we calculated:

(5) *CRT score*: the sum of correct answers (ranging from 0: least reflective to 3: most reflective),

The TIPI is a short-form of the five-factor model of personality that has been validated in several languages (Muck et al., 2007; Oshio et al., 2012; Renau et al., 2013; Chiorri et al., 2014), yielding a further individual difference measure:

(6) *Extraversion*: the two-item average on the Ten Item Personality Inventory (1: most introverted, 7: most extraverted).

Finally, we asked participants to provide the following sociodemographic information: age, gender, native language, nationality, country of residence, educational attainment, religiosity, and political orientation.

Below we report how sample size was determined, all data exclusions, all manipulations, and all measures relevant to the present research question. Participants completed four additional tasks that were unrelated to (and therefore not analyzed for) the current study. This study was reviewed and approved by the Institutional Review Board at the University of Pittsburgh.

### Exclusion Criteria

To identify participants who failed to understand the scenarios, we asked a comprehension question after each scenario. Following the CI scenario, participants were asked whether the scientists in that scenario had to interfere for the murder to take place, and they were excluded if they (incorrectly) responded that the scientists had to interfere. Following the AS scenario, participants were asked whether the murder “was brought about by whatever happened before it” and whether “it had to happen” in a single question, and they were excluded if they (incorrectly) responded that the murder was not brought about by whatever happened before it and that it did not have to happen.

Nearly one in three participants (31%; *n* = 736) failed to comprehend the AS scenario – in contrast to one in eight who misunderstood the CI scenario (*n* = 362). That is, numerous participants ignored or disregarded the instructions stipulating “a universe in which everything that happens is completely brought about by whatever happened before it,” and (incorrectly) reported that the murderer’s decision “was *not* brought about by whatever happened before it” (emphasis added).

This difficulty in conceiving causal determinism was expected in light of recent work on the ‘intrusion of intuitive metaphysics’ (Rose et al., 2015): namely, people’s strong conviction that the actual world is indeterministic hinders the suspension of disbelief required to engage in the AS scenario’s thought experiment.

On one hand, since these participants are construing the universe as *in*deterministic, their responses cannot shed light on their intuitions regarding the conditions for free will and responsibility. Therefore, throughout the section “Results,” we only report analyses of the subsamples who demonstrated comprehension: 2,525 and 1,645 participants for the CI and AS cases, respectively. At the same time, the stark difference in comprehension rates across these two scenarios introduces the risk of an assignment bias, which could jeopardize the comparative analyses reported in section “Comparative Analyses.” To address this concern, in Supplementary Analysis 1 we provide statistical analyses for the full samples, and find that many of our results (including our key finding) are unchanged when we do so.

### Power Analysis

We heed Benjamin et al.’s (2017) recommendation of treating results as statistically significant if their associated *p*-values are lower than 0.005 and as suggestive if they lie between 0.005 and 0.05. A power analysis for small effects (*r* = 0.10) with 80% power revealed a target sample size of 782 participants to uncover suggestive evidence, and 1,325 participants to uncover a significant effect.

### Analytic Approach

Statistical analyses were performed in *R* 3.5.1. In section “Separate Analyses,” we report the results for the AS and CI scenarios separately. Then, in section “Comparative Analyses,” we report comparative analyses of AS and CI data. In every regression and ANCOVA model, we enter age and gender as covariates to mitigate the impact of differences in sample composition across countries. Unless otherwise noted, regressions and ANCOVAs are random coefficients and slopes models, estimated using the *lme4* package (Bates et al., 2015), with participants nested within countries. *P*-values for the fixed effects were calculated using the Satterthwaite approximation in *lmerTest* (Kuznetsova et al., 2017). National and regional comparisons were based on estimated marginal means using the *emmeans* package (Lenth et al., 2019), with significance levels adjusted using the Tukey method for familywise error correction. Mediation and moderated mediation analyses were conducted using the *mediation* package (Tingley et al., 2014) and confidence intervals were derived through 5000 quasi-Bayesian Monte Carlo simulations (see Imai et al., 2010).

Raw data and analysis scripts are available at the following link on the *Open Science Framework*: https://osf.io/t3gzv/.

## Results

### Separate Analyses

Summary statistics are displayed in Table 1 and Supplementary Table 2.

**TABLE 1:** Means, standard deviations, and correlations with confidence intervals.

	**CI**		**AS**		**1**	**2**	**3**	**4**	**5**	**6**
		
	***M***	***SD***	***M***	***SD***						
1. Freedom	0.83	0.38	0.47	0.50	–	0.51^∗∗^[0.47, 0.55]	0.46^∗∗^[0.42, 0.50]	0.44^∗∗^[0.40, 0.48]	−0.16^∗∗^[−0.21, −0.11]	0.09^∗∗^[0.04, 0.13]
2. Control	5.00	2.28	3.31	2.38	0.31^∗∗^[0.27, 0.34]	–	0.50^∗∗^[0.47,0.54]	0.45^∗∗^[0.41,0.49]	−0.17^∗∗^[−0.22, −0.12]	0.11^∗∗^[0.06, 0.16]
3. Blame	5.90	1.64	4.67	2.41	0.39^∗∗^[0.36, 0.42]	0.28^∗∗^[0.24, 0.31]	–	0.80^∗∗^[0.78, 0.81]	−0.12^∗∗^[−0.17, −0.07]	0.04 [−0.01, 0.09]
4. Punishment	5.98	1.44	5.04	2.32	0.44^∗∗^[0.40, 0.47]	0.27^∗∗^[0.24, 0.31]	0.64^∗∗^[0.62, 0.67]	–	−0.10^∗∗^[−0.15, −0.06]	0.03 [−0.02, 0.07]
5. CRT score	1.43	1.19	1.56	1.21	0.04^∗^[0.00, 0.08]	0.00 [−0.04, 0.04]	0.08^∗∗^[0.04, 0.12]	0.09^∗∗^[0.05, 0.13]	–	−0.13^∗∗^[−0.18, −0.08]
6. Extraversion	4.05	1.46	3.99	1.49	−0.01 [−0.05, 0.02]	0.02 [−0.02, 0.06]	0.02 [−0.02, 0.06]	−0.01 [−0.05, 0.03]	−0.06^∗∗^[−0.10, −0.02]	–

**M* and *SD* represent mean and standard deviation, respectively. Values in square brackets indicate the 95% confidence interval for each correlation separately by condition: CI (below diagonal; gray background); AS (above diagonal). ^∗^*p* < 0.05; ^∗∗^*p* < 0.005.*

#### The Counterfactual Intervener (CI) Scenario

Despite lacking alternate possibilities, the perpetrator was seen as acting freely by a significant majority of participants (82%, 95% CI [81%, 84%], binomial test: *p* < 0.001). Moreover, attributions of control (M = 5.00, SD = 2.28), blame (M = 5.90, SD = 1.64), and punishment (M = 5.99, SD = 1.44) were all significantly above the scale midpoint (all one-sample *t*-test *p*s < 0.001). Thus, extending prior research (Miller and Feltz, 2011), participants tended to share Frankfurt’s (1969) intuition that alternate possibilities are not necessary for free will and moral responsibility.

Separate ANCOVAs with *site* as factor revealed substantial differences in ascriptions of control, *F*(20, 2375) = 12.5, ${\text{η}}_{p}^{2}$ = 0.11, blame, *F*(20, 2370) = 18.9, ${\text{η}}_{p}^{2}$ = 0.16, and punishment across sites, *F*(20, 2363) = 9.47, ${\text{η}}_{p}^{2}$ = 0.08, all *p*s < 0.001, while a corresponding logistic regression revealed differences in ascriptions of freedom, χ^2^(*df* = 20) = 217.4, *p* < 0.001. To evaluate whether intuitions varied across world regions, we repeated the above analyses with respondents grouped by world region (rather than by site): Asian respondents ascribed less freedom, control, blame, and punishment than European, Middle Eastern, or American participants, all *p*s < 0.005 (see Table 2). In addition, European participants ascribed less control than either Middle Eastern or American participants, *p*s < 0.001.

**TABLE 2:** Regression-based pairwise comparisons between world regions on each dependent measure.

			**CI**			**AS**		
			***B***	***t***	***p***	***B***	***t***	***p***
		Freedom^∗^	–1.35	–9.88	<0.001	0.04	0.39	0.98
Asia	Europe	Control	–0.39	–3.38	0.004	0.26	1.92	0.22
		Blame	–0.31	–3.73	0.001	0.81	5.97	<0.001
		Punishment	–0.46	–6.35	<0.001	0.64	4.92	<0.001
		Freedom^∗^	–1.17	–4.59	<0.001	0.20	0.95	0.78
Asia	Middle east	Control	–1.31	–6.47	<0.001	0.11	0.45	0.97
		Blame	–0.72	–5.00	<0.001	1.00	4.05	<0.001
		Punishment	–0.66	–5.18	<0.001	1.08	4.57	<0.001
		Freedom^∗^	–2.06	–9.94	<0.001	–0.10	–0.67	0.91
Asia	N. and S. America	Control	–0.95	–7.17	<0.001	–0.02	–0.10	1
		Blame	–0.90	–9.61	<0.001	0.34	1.89	0.23
		Punishment	–0.72	–8.69	<0.001	0.28	1.61	0.37
		Freedom^∗^	–0.18	0.69	0.90	0.15	0.74	0.88
Europe	Middle east	Control	–0.92	–4.69	<0.001	–0.15	–0.61	0.93
		Blame	−0.41	−2.97	−0.016	0.19	0.78	0.86
		Punishment	–0.19	–1.58	0.39	0.44	1.88	0.24
		Freedom^∗^	−0.71	−3.25	0.006	–0.15	–0.97	0.77
Europe	N. and S. America	Control	–0.55	–4.35	<0.001	–0.28	–1.55	0.41
		Blame	–0.59	–6.54	<0.001	−0.46	−2.59	0.047
		Punishment	−0.26	−3.20	0.008	–0.36	–2.08	0.16
		Freedom^∗^	−0.89	−2.89	0.020	–0.3	–1.29	0.57
Middle East	N. and S. America	Control	0.36	1.72	0.31	–0.13	0.47	0.97
		Blame	–0.18	–1.23	0.61	–0.66	–2.40	0.077
		Punishment	–0.06	–0.47	0.97	−0.80	−3.05	0.013

*^∗^coefficients for the Freedom measure are on the logit scale.*

#### The Actual Sequence (AS) Scenario

In the AS scenario, participants were more divided about whether the perpetrator acted freely (47%, 95% CI [44%, 49%]) and they tended to deny control (M = 3.31, SD = 2.38) – with both attributions significantly *below* chance/midpoint, *p*s < 0.005. Still, attributions of blame (M = 4.67, SD = 2.41) and punishment (M = 5.04, SD = 2.32) remained significantly *above* the scale midpoint, *p*s < 0.001. Therefore, in line with previous studies on the problem of free will (Nichols and Knobe, 2007; Miller and Feltz, 2011), people revealed compatibilist responses to both concrete scenarios – whether the agent was characterized as lacking alternate possibilities or sourcehood.

Once again, one-way ANCOVAs entering *site* as a factor revealed differences in perceived control, *F*(19, 1577) = 6.13, ${\text{η}}_{p}^{2}$ = 0.07, blame, *F*(19, 1576) = 6.65, ${\text{η}}_{p}^{2}$ = 0.08, and punishment, *F*(19, 1576) = 5.03, ${\text{η}}_{p}^{2}$ = 0.06, while a corresponding logistic regression revealed differences in ascriptions of freedom, χ^2^(*df* = 19) = 86.9, all *p*s < 0.001.

The ANCOVAs across world regions suggested that Asians ascribed significantly *more* blame and punishment than either Middle Easterners or Europeans, *p*s < 0.001, but no more freedom or control, *p*s > 0.20 (see Table 2). There were few reliable differences between Europeans, Middle Easterners and Americans (only 2 of the 12 pairwise comparisons revealed significant or suggestive differences).

#### Personality and Cognitive Style

Turning to individual differences, we partially replicated the previously reported link between extraversion and free will judgments in the AS case (Feltz and Cokely, 2009, 2019), as shown in Table 1: Extraverts were more likely to ascribe freedom, *r* = 0.09, 95% CI [0.04, 0.13], and they attributed greater control, *r* = 0.11, 95% CI [0.06, 0.16], both *p*s < 0.001. However, they did not attribute greater blame, *r* = 0.04, 95% CI [−0.01, 0.09], or punishment, *r* = 0.02, 95% CI [−0.02, 0.07], than introverts, both *p*s > 0.05.

In line with prior research (Pacini and Epstein, 1999), personality was linked to cognitive style—with extraverts scoring lower on the CRT (α = 0.73) than introverts, *r*(4006) = −0.09 [−0.12, −0.06], *p* < 0.001. We therefore sought to understand whether cognitive style better predicted compatibilist judgments than did extraversion.

Indeed, CRT scores systematically correlated with all four attributions: More reflective participants were less likely to ascribe freedom, *r* = −0.16, 95% CI [−0.21, −0.11], they attributed less control, *r* = −0.17, 95% CI [−0.22, −0.13], and also viewed the perpetrator as less worthy of blame, *r* = −0.12, 95% CI [−0.17, −0.07], or punishment, *r* = −0.10, 95% CI [−0.15, −0.06], all *p*s < 0.001 – even after controlling for differences in extraversion.^2^ In sum, we successfully replicated some effects of extraversion – but, overall, cognitive style revealed a more robust association with judgments of free will and moral responsibility than did personality (see also the Supplementary Analysis 1).

### Comparative Analyses

The AS and CI scenarios differ in a few respects; yet, the most salient philosophical difference concerns the question of ultimate sourcehood (Pereboom, 2001). Neither perpetrator could have done otherwise, but they differ with regards to sourcehood: The perpetrator in the AS case lacks sourcehood, while the perpetrator in the CI case *is* the ultimate source of his actions. Does the presence of sourcehood promote the perception that an agent acted freely and should be held morally responsible? To shed light on this question, we compare responses to both thought experiments below.

#### Cultural Differences

To begin with, we conducted a logistic regression (for the dichotomous freedom measure) and two-way ANCOVAs (for the control, blame, and punishment measures), entering scenario, site, and the scenario × site interaction. These analyses revealed main effects of scenario, *F*s > 271, and site, *F*s > 6, on all dependent measures, all *p*s < 0.001. The main effect of scenario indicated that participants were more likely to ascribe freedom *OR* = 6.99, 95% CI [5.75, 8.55], and they ascribed greater control *B* = 1.56, 95% CI [1.41, 1.73], blame *B* = 1.18, 95% CI [1.05, 1.32], and punishment *B* = 0.93, 95% CI [0.80, 1.06], to the perpetrator in the CI condition than in the AS condition, all *p*s < 0.001.

We also observed a scenario × site interaction in every model: freedom, χ^2^(19, *N* = 3987) = 174.8; control, *F*(19, 4005) = 8.11, η*_*p*_*^2^ = 0.04; blame, *F*(19, 3999) = 8.94, η*_*p*_*^2^ = 0.04; and punishment, *F*(19, 3992) = 7.59, η*_*p*_*^2^ = 0.04, all *p*s < 0.001 – pointing toward cultural differences in the import of sourcehood for free will.

Tests of the simple effect of scenario in each site revealed systematic regional variation, dovetailing with previously reported East-West differences (Miller, 1984; Morris and Peng, 1994; Choi et al., 1999): Throughout American, European, and Middle Eastern countries, participants overwhelmingly judged that the perpetrator in the CI case acted more freely, exercised greater control, and also deserved greater blame and harsher punishment than the perpetrator in the AS case (*p*s < 0.005 with five exceptions).^3^

In contrast, among most Asian countries, we found no corresponding effect of scenario: Rather, Asian participants tended to assign comparable blame and punishment to both perpetrators, i.e., in mainland China, Hong Kong, India, and Indonesia, *p*s > 0.05. Moreover, we failed to find any difference in judgments about whether the agent was in control (for mainland China, Hong Kong, India, and South Korea, *p*s > 0.250) or acted freely (for mainland China, Hong Kong, and Indonesia, *p*s > 0.250).

**FIGURE 1:**
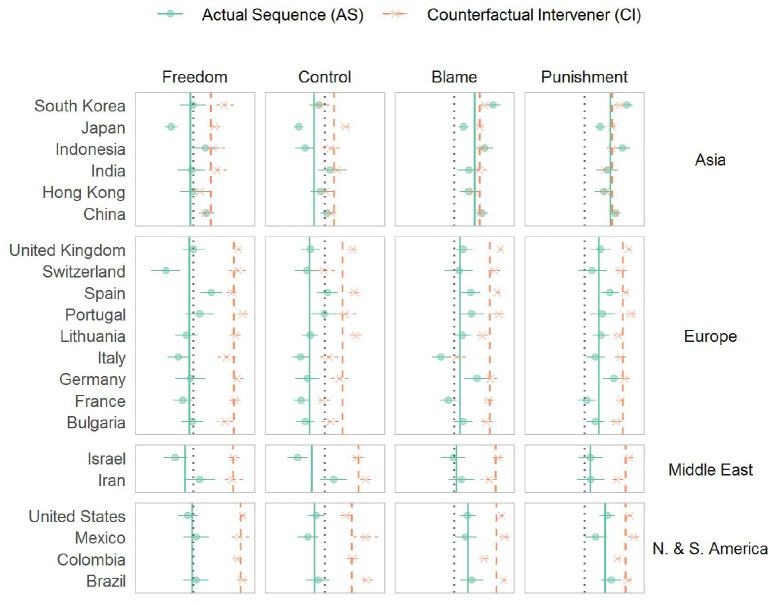
Mean ascriptions of freedom, control, blame and punishment by scenario. Observed means for each location (/site) and their 95% confidence intervals are plotted on the *x*-axis. A dotted vertical line represents the scale midpoint, and world region means are displayed using solid and dashed vertical lines.

A multivariate meta-analysis with site and dependent measure as cross-classified random effects confirmed this pattern of cultural moderation: There was substantial heterogeneity across world regions, QM(*df* = 3) = 12.5, *p* = 006. Specifically, though no differences emerged between Europe, North and South America, and the Middle East, *z*s < 1, *p*s > 0.50, Asia revealed a reduced effect across dependent measures and sites, *r* = −0.23, 95% CI [−0.40, −0.06] *z* = −2.66, *p* = 0.008 (see Figure 1).

#### The Role of Control Across Cultures

Attribution theory (Heider, 1958; Weiner, 1985) predicts that the greater tendency to hold agents *with* sourcehood morally responsible (e.g., in the CI condition) arises from the perception that the agent exercised greater control over their behavior (see also Monroe et al., 2014; Monroe and Malle, 2019). Specifically, the CI condition, in which the counterfactual intervener did *not* have to interfere, may invite an *intrinsic* attribution – i.e., for participants to see the murderer’s actions as a reflection of his evil character. Meanwhile, the AS case – in which behavior is ultimately explained by antecedent causes and the laws of nature – invites an *extrinsic* attribution, and the corresponding assessment that the agent should not be held morally responsible.

To evaluate this prediction, we averaged blame and punishment ratings to form an index of *moral responsibility* (Cronbach’s α = 0.86), and tested whether attributions of control statistically mediate the difference in moral responsibility ascription across scenarios. As with all of our prior models, both our mediator and outcome models incorporated age and gender as covariates:

$$control=a\times scenario+x_{1}\times age+x_{2}\times gender+i\;(\mathrm{mediator}\mathrm{model})$$

$$m\,o\,r\,a\,l\,r\,e\,s\,p\,o\,n\,s\,i\,b\,i\,l\,i\,t\,y=b\times c\,o\,n\,t\,r\,o\,l+c\times s\,c\,e\,n\,a\,r\,i\,o+y_{1}\times agey_{2}\times gender+i\left(\mathrm{outcome}\mathrm{model}\right)$$

We calculated the *proportion of the total effect that is mediated* (i.e., *prop. mediated*). The total effect equals the sum of the direct effect (expressed by coefficient *c* in the outcome model), and the indirect effect via the mediator (which amounts to *a* × *b*, the product of the effect of scenario in the mediator model and the effect of judged control in the outcome model); therefore:

p⁢r⁢o⁢p.m⁢e⁢d⁢i⁢a⁢t⁢e⁢d=a⁢b/(a⁢b+c)

Averaging the indirect effect estimate, *ab*, across 5000 Monte Carlo simulations yields the average causally mediated effect, *ACME*. As expected, control attributions mediated the effect of scenario on moral responsibility judgments among American (*n* = 726; ACME = 0.72, 95% CI [0.47, 0.98]; prop. mediated = 0.52, 95% CI [0.31, 0.98]), European (*n* = 1711; ACME = 0.57, 95% CI [0.44, 0.72]; prop. mediated = 0.41, 95% CI [0.29, 0.59]), and Middle Eastern (*n* = 255; ACME = 1.34, 95% CI [0.56, 2.17], prop. mediated = 0.66, 95% CI [0.43, 1.15]) participants; *p*s < 0.005. In each of these world regions, people viewed the perpetrator in the CI scenario as having exercised greater control than the perpetrator in the AS scenario. This difference in perceived control accounted for part of the effect of scenario on moral responsibility evaluations – as predicted by theories of attributional reasoning.

In contrast, the mediation model failed to predict the data obtained throughout Asian countries (*n* = 1302; ACME = −0.08, 95% CI [−0.01, 0.17], *p* = 0.092; prop. mediated = −0.11, 95% CI [−5.57, 5.06], *p* = 0.86).^4^ This result dovetails with abundant evidence that Asian cultures differ in their explanatory style (Miller, 1984; Morris and Peng, 1994; Choi et al., 1999; Chiu et al., 2000; Feinberg et al., 2019). A wide literature reveals that Asian individuals gravitate toward *situational* (and not dispositional) attributions – which could explain why they evaluate these two crimes in a very similar way – i.e., by viewing both perpetrators as subject to extrinsic pressures that drove them to act.

#### Moderated Mediation Model

**FIGURE 2:**
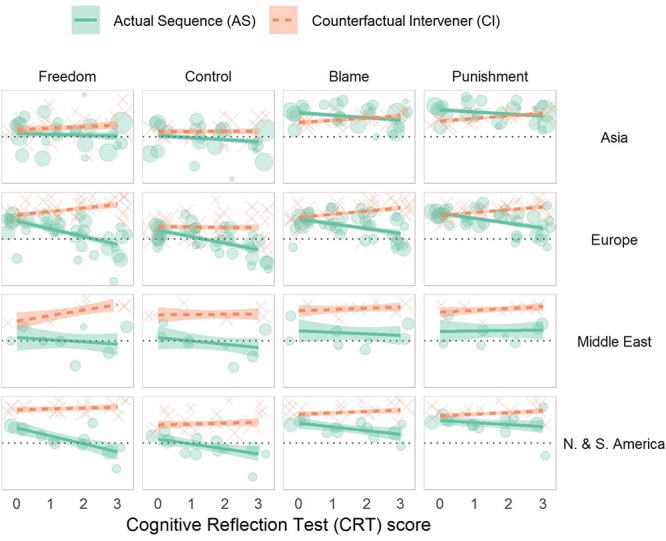
Mean ascriptions by scenario and CRT score. We plot observed means for each dependent measure, grouped by location (/site) and score on the CRT. Point size is proportional to the number of observations. A dotted horizontal line represents the scale midpoint, and linear trends by world region are displayed using solid and dashed lines.

As demonstrated in section “Personality and Cognitive Style,” reflective participants ascribed less moral responsibility to the perpetrator in the AS case. No corresponding association was shown for the CI case – if anything, reflective participants ascribed slightly *more* moral responsibility to the perpetrator, when merely lacking alternate possibilities (see Table 1 and Figure 2). Furthermore, the mitigating effect of cognitive reflection emerged only among participants who grasped determinism, whereas the associations with personality generalized to those who misunderstood the AS scenario and perceived the perpetrator’s behavior as causally *in*determined (see Supplementary Analysis 1).

Taken together, these results may indicate that cognitive reflection supports the conclusion that ultimate sourcehood is a condition for free will and moral responsibility (see, e.g., Pereboom, 2001). Perhaps, reflective individuals are more likely to engage in dispositionist reasoning, and ascribe control when the agent in question is seen as the ultimate source of her behavior.

Indeed, reflective participants perceived a greater difference in freedom, OR = 2.08, 95% CI [1.62, 2.67], *z* = 5.76, and control, *B* = 0.47, 95% CI [0.30, 0.64], *t* = 5.42, across scenarios; *p*s < 0.001.^5^ Meanwhile, in the same models, the interaction between extraversion and scenario was far from significant (freedom: OR = 0.92, 95% CI [0.77, 1.11], *z* = −0.88, *p* = 0.38; control *B* = −0.14, 95% CI [−0.31, 0.04], *t* = −1.53, *p* = 0.13).

Does the greater difference in *control* ascriptions among reflective participants explain why they tended to selectively exculpate the agent in the AS scenario on measures of *moral responsibility*? To assess this hypothesis, we entered CRT score and the CRT×scenario interaction into the mediator and outcome models defined in section “The Role of Control Across Cultures.” We can now ask whether the magnitudes of *c* (the direct effect) and *ab* (the indirect effect) reliably depend on participants’ CRT score. If cognitive reflection underlies dispositionist reasoning, we should observe that the indirect effect via control is larger among reflective (than unreflective) participants.

**FIGURE 3:**
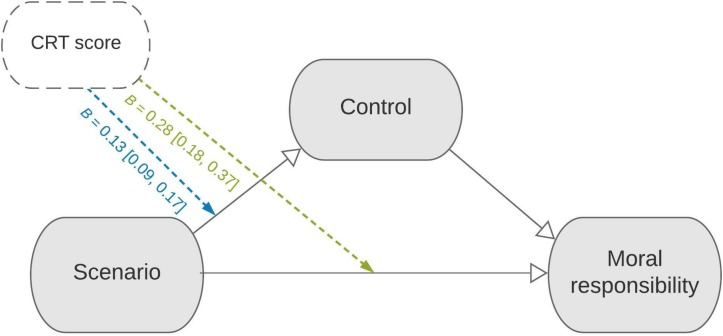
Moderated mediation diagram. Cognitive style moderates the indirect and direct effects of scenario on attributions of moral responsibility via perceived control (both *p*s < 0.005).

Indeed, as depicted in Figure 3, CRT score moderated the indirect effect of scenario on moral responsibility, *B* = 0.13, 95% CI [0.09, 0.17], *p* < 0.001. In other words, reflective individuals selectively ascribed less moral responsibility to the agent in the AS scenario than did intuitive individuals, and this effect was mediated by differences in the degree to which they viewed the agent as exercising control.

In several Asian countries, participants did not perceive a difference in control across scenarios – which we interpreted in light of their documented emphasis on situationist explanations. Might reflection therefore play a qualitatively distinct role in attitudes toward the free will problem in these cultural contexts? To the contrary, even throughout Asian countries, cognitive reflection supported incompatibilist responses to the AS case^6^ (freedom *OR* = 1.33, 95% CI [1.06, 1.67], *z* = 2.43, *p* = 0.015; control *B* = 0.42, 95% CI [0.18, 0.66], *t* = 3.38, *p* < 0.001) and moderated the indirect (ACME = 0.09, 95% CI [0.04, 0.14], *p* < 0.001) effect of scenario on moral responsibility.

In sum, reflective participants ascribed less freedom and control – and derivatively, assigned reduced moral responsibility – to the perpetrator in the AS condition (but not in the CI condition). This effect of cognitive reflection accounted for previously reported associations with extraversion, and emerged even throughout Asian cultures that tended not to sharply distinguish the AS and CI scenarios. Importantly, failure to comprehend the scenarios is unlikely to explain these results, since we excluded participants who failed our comprehension checks in each condition.

Though not predicted by our theory, cognitive reflection also moderated the *direct* effect of scenario, *B* = 0.28, 95% CI [0.18, 0.37], *p* < 0.001, perhaps because incompatibilist reasoning is not based exclusively on assessments of agent control, but also on constructs we did not observe in this study, such as beliefs about the agent’s genuine or second-order desires (Frankfurt, 1969; Cullen, 2018).

## Discussion

At the aggregate level, we found that participants blamed and punished agents whether they only lacked alternate possibilities (Miller and Feltz, 2011) or whether they also lacked sourcehood (Nahmias et al., 2005; Nichols and Knobe, 2007). Thus, echoing early findings, laypeople did not take alternate possibilities or sourcehood as necessary conditions for free will and moral responsibility.

Yet, our study also revealed a dramatic cultural difference: Throughout the Americas, Europe, and the Middle East, participants viewed the perpetrator with sourcehood (in the CI scenario) as freer and more morally responsible than the perpetrator without sourcehood (in the AS scenario). Meanwhile, South and East Asian participants evaluated both perpetrators in a strikingly similar way. We interpreted these results in light of cultural variation in dispositional versus situational attributions (Miller, 1984; Morris and Peng, 1994; Choi et al., 1999; Chiu et al., 2000). From a dispositionist perspective, participants may be especially attuned to the absence of sourcehood: When an agent is the source of their action, people may naturally conjure dispositionist explanations that refer to her goals, desires (e.g., because “she wanted a new life”) or character (e.g., because “she is ruthless”). In contrast, when actions result from a causal chain originating at the beginning of the universe, explanations of this sort – implying sourcehood – seem particularly unsatisfactory and incomplete. In contrast, from a situationist perspective, whether the agent could be seen as the source of her action may be largely irrelevant: Instead, a situationist may think of others’ behavior as the product of extrinsic pressures – from momentary upheaval, to the way they were raised, social norms or fate – and thus perceive both agents, in the CI and AS cases, as similar in matters of free will and moral responsibility.

Throughout American, European, and Middle Eastern cultures, dispositionist explanations seemed to prevail: Participants tended to hold the perpetrator with sourcehood morally responsible, but ascribed less moral responsibility to the perpetrator without sourcehood – and this effect was even larger among participants who exhibited greater cognitive reflection. Meanwhile, consistent with past evidence (Miller, 1984; Morris and Peng, 1994; Choi et al., 1999; Chiu et al., 2000), Asian cultures showed signs of *both* dispositionist and situationist reasoning: Overall, they tended not to distinguish between agents with and without sourcehood, suggesting a default preference for situationist explanations. Still, as with other world regions, reflective participants throughout Asia were more likely to selectively exculpate the agent lacking sourcehood. It could be that Asians tended *intuitively* toward situationist explanations, but were more likely to conjure alternative dispositionist explanations upon further reflection.

Our study also shed new light on the understanding of individual differences in compatibilist beliefs. Several studies previously reported that extraverts and introverts differ in their assessments of whether free will and determinism are compatible (Feltz and Cokely, 2009; Schulz et al., 2011), but yielded limited insight into why. Building on evidence that extraverts and introverts differ in their cognitive style, we found that a tendency toward cognitive reflection largely subsumed the previously reported effect of personality. This finding helps us interpret extraverts’ and introverts’ divergent attitudes toward the free will problem as the result of associated differences in trait reflectivity: In the wake of a heinous crime, we are immediately motivated to condemn the perpetrator, prior to reasoning about whether she exercised free will or control (Nichols and Knobe, 2007). While reflective individuals may evaluate the implications of determinism for free will, concluding that sourcehood (but not alternate possibilities) is a condition for free will, less reflective participants may readily attribute moral responsibility, and even ascribe free will, motivated by their initial punitive drive (Clark et al., 2014, 2017).

### Limitations

First, throughout our comparative analyses we emphasized sourcehood as the primary factor driving the difference between scenarios. Yet, the AS and CI scenarios differed in several other ways (e.g., the length of the vignette, the perpetrator’s motives), which could have contributed to the greater free will and moral responsibility ascriptions in the CI case.

Second, participants faced acute comprehension difficulties in the AS scenario – introducing potential assignment bias into the comparative analyses. Even though the stimuli dictated that “everything that happens is completely brought about by whatever happened before it,” nearly a third of participants denied that the murderer’s decision had been brought about by prior events. This high rate of comprehension failure has been documented in previous studies, and attributed to overpowering indeterministic assumptions (Rose et al., 2015) as well as to a morally motivated denial of determinism (Clark et al., 2019). It may also have been aggravated by the complexity with which determinism was introduced in our vignette.

The results of our Supplementary Analysis 1 help to alleviate both concerns. (1) The effect of cognitive reflection fully reversed among participants who misunderstood the AS scenario: Reflective participants who incorrectly construed the scenario as *in*deterministic ascribed *more* free will and moral responsibility than did intuitive participants – in line with our favored interpretation that (perceived) sourcehood promotes attributions of free will and moral responsibility under cognitive reflection. (2) When re-running the comparative analyses based on scenario assignment, regardless of comprehension, the scenario difference in free will and moral responsibility ascriptions remained significant – suggesting that assignment bias alone did not produce the difference between scenarios. Still, in future research, we aim to employ maximally matched stimuli, and pre-test instructions that facilitate comprehension and balance exclusion rates across conditions.

Third, because our study did not specifically measure dispositional and situational explanatory styles, we submit that the observed difference between Asia and other world regions could instead be driven by other dimensions of cultural psychology, such as individualism versus collectivism (Triandis, 1995), the prevailing self-concept (Markus and Kitayama, 1991; Heine and Hamamura, 2007), or analytic versus holistic thinking (Nisbett et al., 2001; Feinberg et al., 2019).

## Conclusion

After more than a decade of research on the cognitive science of free will, we have learned that laypeople’s beliefs vary systematically, depending on how (Nichols and Knobe, 2007) and who (Feltz and Cokely, 2009; Schulz et al., 2011) you ask. Yet, prior research on judgments of free will suffered from an important limitation: Most past studies relied on small and homogeneous North American samples (but see Sarkissian et al., 2010). The present work addressed this limitation by surveying thousands of participants in twenty countries and sixteen languages.

In so doing, our study documented individual and cultural variations in views about the problem of free will. First, in most world regions, people ascribe greater free will to an agent who merely lacks alternate possibilities (in a Frankfurt case) than to one who also lacks sourcehood (in a deterministic universe). By contrast, being the source of one’s actions is not particularly salient throughout Asian cultures – perhaps due to the greater tendency toward situationist over dispositionist explanations of behavior. Second, reflective participants were more likely to treat ultimate sourcehood as a condition for free will, even in predominantly situationist cultures. Importantly, the cultural difference – i.e., between situationist and dispositionist cultures – was not the result of variations in cognitive style. Rather, culture and reflectivity independently contributed to lay judgments about the conditions for free will and moral responsibility.

In closing, our findings have certain implications for philosophical methodology: Debates about the problem of free will have generally focused on establishing the predominant intuition as an element in empirically informed argumentation. Given the present evidence of substantial individual and cultural variability, this approach seems misguided (Machery, 2017). Clearer insights into the free will problem may instead be gleaned by understanding the psychological and cultural bases of disagreement concerning the tension between determinism and free will.

## Data Availability Statement

The datasets generated for this study can be found in the Open Science Framework: https://osf.io/t3gzv/.

## Ethics Statement

The studies involving human participants were reviewed and approved by the University of Pittsburgh. The patients/participants provided their written informed consent to participate in this study.

## Author Contributions

EM, SS, and DR designed the study. IH analyzed and interpreted the data under the supervision of EM and CO. IH drafted the manuscript. EM, SS, DR, CO, EB, FC, and PS critically revised the manuscript. All authors were involved in data collection, and approved the final manuscript for publication.

## Conflict of Interest

The authors declare that the research was conducted in the absence of any commercial or financial relationships that could be construed as a potential conflict of interest.

## Footnotes

1 Due to a technical problem, the AS scenario was not administered in Colombia.

2 When simultaneously entering both CRT scores and extraversion ratings as predictors, we find that CRT scores predict judgments of freedom (β = −0.33, 95% CI [−0.48, −0.18], *z* = −4.44, *p* < 0.001), control (β = −0.43, 95% CI [−0.57, −0.30], *t* = −6.43), blame (β = −0.27, 95% CI [−0.43, −0.11], *t* = −3.48, *p* = 0.004), and punishment (β = −0.24, 95% CI [−0.40, −0.08], *t* = 3.04, *p* = 0.007). Extraversion independently predicted judgments of freedom (β = 0.12, 95% CI [0.00, 0.24], *z* = −1.96, *p* = 0.042) and control (β = 0.19, 95% CI [0.06, 0.32], *t* = 2.86, *p* = 0.007), but not blame (β = 0.05, 95% CI [−0.08, 0.18], *t* = 0.79, *p* = 0.43) or punishment (β = 0.03, 95% CI [−0.12, 0.17], *t* = 0.40, *p* = 0.69).

3 Only five out of 56 tests revealed *p-*values > 0.005, a result that might be expected by chance alone: Germany-blame *p* = 0.057, Germany-punishment *p* = 0.064. Italy-blame *p* = 0.026. Switzerland-control *p* = 0.061. Portugal-control *p* = 0.020. As seen in Figure 1, every effect trended in the predicted direction, *p*s < 0.10.

4 Note that the proportion of the effect that is mediated can be negative when the direct and indirect effects have opposite signs.

5 Corresponding effects emerged for blame and punishment: blame *B* = 0.36, 95% CI [0.20, 0.53], *t* = 4.36; punishment *B* = 0.32, 95% CI [0.18, 0.46], *t* = 4.42; both *p*s < 0.001.

6 Looking exclusively at Asian countries, CRT scores predicted freedom *r* = −0.12, *p* = 0.014; control *r* = −0.14, *p* = 0.006; blame *r* = −0.13, *p* = 0.009; and punishment *r* = −0.15, *p* = 0.004, attributions in the AS condition.
