# Supplementary material for: For Whom Does Determinism Undermine Moral Responsibility? Surveying the Conditions for Free Will Across Cultures
Source: Front Psychol. 2019 Nov 5;10:2428. doi: 10.3389/fpsyg.2019.02428 (PMC6848273; doi:10.3389/fpsyg.2019.02428)
Supplement: Supplementary file 1 [file Table_1.DOCX]

Supplementary Material

# Supplementary Table 1

Sample characteristics by location.

|  | ***N*** | **Age**  **M (SD)** | **Gender**  **(% women)** | **n_AS_ (excl.%)** | **n_CI_**  **(excl. %)** | **Students** | **Method** | **Payment** | **Language** | **Recruitment** |
| --- | --- | --- | --- | --- | --- | --- | --- | --- | --- | --- |
| **Asia** |  |  |  |  | | | | |  |  |
| China (mainland) | 541 | 24.1 (6.25) | 50% | 166 (39%) | 202 (25%) | S, NS | P, W | V, C | Chinese | Local collaborator |
| Hong Kong | 195 | 20.1 (2.18) | 25% | 60 (38%) | 93 (5%) | S | W | C | Chinese | Local collaborator |
| India | 200 | 22.2 (6.09) | 38% | 56 (44%) | 85 (15%) | S | P | V | Bengali | Local collaborator |
| Indonesia | 199 | 19.6 (1.19) | 51% | 54 (45%) | 66 (34%) | S | P | C | Indonesian | Local collaborator |
| Japan | 511 | 28.8 (13.8) | 47% | 204 (24%) | 212 (12%) | S, NS | P, W | V, C | Japanese | Local collaborator |
| South Korea | 173 | 39.4 (11.9) | 51% | 63 (31%) | 69 (16%) | NS | W | C | Korean | Research firm |
| **Europe** |  |  |  |  | | | | |  |  |
| Bulgaria | 247 | 30.8 (10.4) | 26% | 95 (23%) | 114 (8%) | S, NS | W | V | Bulgarian | Local collaborator |
| France | 368 | 42.0 (16.7) | 58% | 113 (42%) | 152 (12%) | NS | W | V, C | French | Bilendi |
| Germany | 196 | 52.3 (13.5) | 45% | 47 (52%) | 87 (11%) | NS | W | C | German | Bilendi |
| Italy | 197 | 21.4 (2.39) | 56% | 87 (13%) | 89 (8%) | S | P | V | Italian | Bilendi |
| Lithuania | 302 | 27.5 (11.3) | 31% | 88 (39%) | 141 (11%) | S, NS | P | V | Lithuanian | Local collaborator |
| Portugal | 121 | 34.3 (13.1) | 50% | 52 (12%) | 58 (6%) | S | P | V | Portuguese | Local collaborator |
| Spain | 400 | 40.0 (11.2) | 59% | 81 (39%) | 224 (16%) | NS | W | C | Spanish | Bilendi |
| Switzerland | 100 | 25.9 (9.36) | 33% | 38 (19%) | 47 (11%) | S | P, W | V, C | French | Local collaborator |
| United Kingdom | 402 | 54.1 (13.9) | 47% | 84 (34%) | 238 (13%) | NS | W | C | English | Bilendi |
| **Middle East** |  |  |  |  | | | | |  |  |
| Iran | 100 | 29.4 (8.55) | 48% | 45 (10%) | 44 (12%) | NS | P | V | Persian | Local collaborator |
| Israel | 195 | 37.9 (12.8) | 46% | 67 (18%) | 109 (4%) | S | W | V | Hebrew | Local collaborator |
| **North, Central & South America** |  |  |  |  | | | | |  |  |
| Brazil | 160 | 21.3 (6.85) | 43% | 69 (14%) | 74 (8%) | S | P | V | Portuguese | Local collaborator |
| Colombia | 170 | 20.9 (4.43) | 74% | - | 156 (8%) | NS | R | C | Spanish | Local collaborator |
| Mexico | 119 | 30.1 (13.2) | 50% | 66 (27%) | 27 (7%) | NS | P | V | Spanish | Local collaborator |
| United States | 372 | 34.4 (10.7) | 44% | 110 (11%) | 238 (4%) | NS | W | C | English | Amazon MTurk |

***N***: pre-exclusion sample size; ***Age***: mean (standard deviation) in years; ***Gender***: percentage of women; ***n*_AS_**, ***n*_CI_**: post-exclusion sample size for each scenario (comprehension failure percentages); ***Students***: S = students, NS = non-students; ***Method***: P = pen-and-paper, W = Web-based, R = Read; ***Payment***: V = volunteers, C = compensated; ***Recruitment***: Local collaborator = a project collaborator recruited participants (e.g., at a university, by circulating a survey link), Bilendi = an external market research firm ([www.bilendi.com](http://www.bilendi.com/)) collected responses from their national panels; Research firm = a local survey research firm collected responses from their panel. Amazon MTurk = Respondents were recruited from [www.mturk.com](http://www.mturk.com), a popular online crowdsourcing website.

# Supplementary Table 2

Summary statistics by scenario and location.

|  |  |  | Freedom | | Control | | | Blame | | | Punishment | | |
| --- | --- | --- | --- | --- | --- | --- | --- | --- | --- | --- | --- | --- | --- |
| Continent | Location | Condition | *N* | *p̂* | *N* | *M* | *SD* | *N* | *M* | *SD* | *N* | *M* | *SD* |
| Asia | China (mainland) | AS | 166 | 0.62 | 166 | 4.11 | 2.35 | 166 | 5.54 | 2.04 | 165 | 5.70 | 1.94 |
|  | China (mainland) | CI | 202 | 0.60 | 201 | 4.33 | 2.15 | 201 | 5.43 | 1.36 | 201 | 5.70 | 1.38 |
|  | Hong Kong | AS | 60 | 0.50 | 60 | 3.75 | 2.06 | 60 | 4.80 | 1.98 | 60 | 5.07 | 1.96 |
|  | Hong Kong | CI | 93 | 0.56 | 93 | 4.03 | 2.17 | 93 | 5.06 | 1.69 | 93 | 5.19 | 1.62 |
|  | India | AS | 56 | 0.48 | 56 | 4.30 | 2.61 | 56 | 4.80 | 2.43 | 56 | 5.25 | 2.31 |
|  | India | CI | 85 | 0.72 | 85 | 4.66 | 2.54 | 85 | 5.35 | 2.14 | 85 | 5.11 | 2.05 |
|  | Indonesia | AS | 54 | 0.61 | 54 | 2.93 | 2.02 | 54 | 5.69 | 1.81 | 54 | 6.09 | 1.59 |
|  | Indonesia | CI | 66 | 0.68 | 66 | 4.29 | 2.27 | 66 | 5.55 | 1.66 | 66 | 5.50 | 1.36 |
|  | Japan | AS | 204 | 0.29 | 203 | 2.56 | 1.85 | 204 | 4.52 | 2.11 | 204 | 4.88 | 2.07 |
|  | Japan | CI | 212 | 0.69 | 212 | 5.14 | 1.87 | 212 | 5.41 | 1.62 | 212 | 5.53 | 1.46 |
|  | South Korea | AS | 63 | 0.49 | 63 | 3.68 | 2.20 | 63 | 6.16 | 1.62 | 63 | 6.35 | 1.23 |
|  | South Korea | CI | 69 | 0.78 | 69 | 3.71 | 2.24 | 69 | 5.68 | 1.39 | 69 | 5.93 | 1.19 |
| Europe | Bulgaria | AS | 95 | 0.48 | 95 | 2.93 | 2.43 | 95 | 4.48 | 2.60 | 95 | 4.60 | 2.57 |
|  | Bulgaria | CI | 114 | 0.80 | 114 | 4.31 | 2.50 | 114 | 5.76 | 1.78 | 114 | 5.74 | 1.72 |
|  | France | AS | 113 | 0.40 | 113 | 2.71 | 2.18 | 113 | 3.68 | 2.52 | 113 | 4.14 | 2.56 |
|  | France | CI | 151 | 0.89 | 152 | 3.86 | 2.47 | 152 | 5.91 | 1.65 | 151 | 5.99 | 1.46 |
|  | Germany | AS | 47 | 0.47 | 47 | 3.04 | 2.33 | 46 | 5.26 | 2.41 | 47 | 5.62 | 2.08 |
|  | Germany | CI | 84 | 0.90 | 87 | 4.79 | 2.43 | 87 | 5.99 | 1.89 | 86 | 6.19 | 1.42 |
|  | Italy | AS | 87 | 0.36 | 87 | 2.68 | 2.16 | 86 | 3.27 | 2.39 | 87 | 4.59 | 2.48 |
|  | Italy | CI | 89 | 0.81 | 88 | 4.22 | 2.47 | 89 | 4.11 | 2.57 | 89 | 5.97 | 1.60 |
|  | Lithuania | AS | 88 | 0.43 | 88 | 3.20 | 2.18 | 88 | 4.45 | 2.26 | 88 | 4.91 | 2.14 |
|  | Lithuania | CI | 139 | 0.89 | 141 | 5.69 | 1.98 | 141 | 5.56 | 1.83 | 141 | 5.87 | 1.60 |
|  | Portugal | AS | 52 | 0.56 | 52 | 3.96 | 2.61 | 52 | 4.96 | 2.57 | 50 | 5.00 | 2.56 |
|  | Portugal | CI | 57 | 0.96 | 58 | 5.10 | 2.44 | 58 | 6.47 | 1.19 | 58 | 6.55 | 1.06 |
|  | Spain | AS | 81 | 0.67 | 81 | 4.14 | 2.64 | 80 | 4.91 | 2.42 | 80 | 5.41 | 2.27 |
|  | Spain | CI | 222 | 0.86 | 224 | 5.62 | 2.14 | 222 | 6.38 | 1.32 | 216 | 6.24 | 1.47 |
|  | Switzerland | AS | 38 | 0.24 | 38 | 3.03 | 2.14 | 38 | 4.26 | 2.43 | 38 | 4.42 | 2.45 |
|  | Switzerland | CI | 47 | 0.91 | 46 | 3.93 | 2.23 | 47 | 6.21 | 1.23 | 47 | 5.98 | 1.19 |
|  | United Kingdom | AS | 84 | 0.50 | 83 | 3.22 | 2.55 | 84 | 4.50 | 2.46 | 83 | 4.89 | 2.49 |
|  | United Kingdom | CI | 238 | 0.92 | 238 | 5.51 | 2.07 | 234 | 6.55 | 1.15 | 232 | 6.42 | 1.16 |
| Middle East | Iran | AS | 45 | 0.56 | 45 | 4.49 | 2.53 | 45 | 4.40 | 2.46 | 45 | 4.36 | 2.58 |
|  | Iran | CI | 44 | 0.86 | 44 | 6.23 | 1.12 | 44 | 5.93 | 1.48 | 44 | 5.77 | 1.26 |
|  | Israel | AS | 67 | 0.33 | 67 | 2.49 | 2.36 | 66 | 3.94 | 2.82 | 67 | 4.33 | 2.79 |
|  | Israel | CI | 109 | 0.87 | 109 | 5.72 | 2.15 | 109 | 6.43 | 1.43 | 109 | 6.47 | 1.28 |
| N., C., & S. America | Brazil | AS | 69 | 0.52 | 69 | 3.62 | 2.66 | 69 | 4.97 | 2.65 | 69 | 5.48 | 2.39 |
|  | Brazil | CI | 74 | 0.96 | 74 | 6.28 | 1.64 | 73 | 6.71 | 0.81 | 74 | 6.38 | 0.98 |
|  | Colombia | CI | 156 | 0.91 | 156 | 5.51 | 1.99 | 156 | 5.64 | 1.59 | 156 | 5.79 | 1.30 |
|  | Mexico | AS | 65 | 0.52 | 66 | 3.06 | 2.46 | 66 | 4.64 | 2.56 | 66 | 4.62 | 2.50 |
|  | Mexico | CI | 27 | 0.93 | 27 | 6.22 | 1.93 | 27 | 6.78 | 0.70 | 27 | 6.70 | 0.78 |
|  | United States | AS | 110 | 0.45 | 109 | 3.52 | 2.45 | 110 | 4.74 | 2.36 | 110 | 5.25 | 2.25 |
|  | United States | CI | 238 | 0.96 | 238 | 5.15 | 2.32 | 238 | 6.62 | 0.95 | 238 | 6.48 | 1.07 |

N: sample size; *p̂*: proportion; M: mean; SD: standard deviation.

# Supplementary Analyses

Here we report statistical analyses of the full sample, including participants who failed the comprehension question. The perpetrator in the CI scenario was seen as acting freely by a significant majority of participants (77%, 95% CI [75%, 78%]), binomial test: *p* < .001, and attributions of control (M = 4.84, SD = 2.29), blame (M = 5.70, SD = 1.77), and punishment (M = 5.79, SD = 1.59) were all significantly above the scale midpoint—according to one-sample *t*-tests, *p*s < .001.

In the AS scenario, we observed a couple of noteworthy differences when including participants who mistook the universe as indeterministic. In these full-sample analyses, a small majority of participants viewed the perpetrator as acting freely (54%, 95% CI [52%, 56%], *p* < .001), and they were divided about whether he exercised control (M = 3.93, SD = 2.38, *p* = .17). Still, attributions of blame (M = 5.13, SD = 2.28) and punishment (M = 5.45, SD = 2.14) remained significantly above the scale midpoint, *p*s < .001.

Critically, the primary comparative analysis of the difference in attributions across scenarios remained statistically significant across all four dependent measures (*β_Freedom_* = -1.22; *β_Control_* = -0.90; *β_Blame_* = -0.63; *β_Punishment_* = -0.43), *p*s < .001—ruling out the concern that our exclusion criteria alone yielded the observed difference between scenarios.

## Personality and cognitive style

When looking at the entire sample, extraverts scored lower on the CRT than did introverts, *r*(5046) = -.09, 95% CI [-.11, -.06], *p* < .001.

Examining the effects of extraversion and cognitive style among participants who failed the comprehension question revealed a noteworthy dissimilarity, relative to participants who passed it. Extraversion remained weakly, yet positively, correlated with attributions in the AS case, .00 < *r*s < .09. The main effects of extraversion on control, blame, and punishment were suggestive, *F*s > 5, *p*s < .05, whereas its interactions with comprehension status were not, *F*s < 1, *p*s > .50. In other words, extraverts tended to ascribe slightly greater free will and moral responsibility, regardless of whether they perceived the behavior as causally determined or indetermined.

For the CRT, the direction of the correlations reversed. Among participants who perceived the universe as indeterministic, cognitive reflection *positively* predicted ascriptions, .04 < *r*s < .14 (whereas it was a negative predictor among participants who passed the comprehension check). In fact, significant interactions between comprehension status and cognitive style emerged for all measures, *F*s > 9, *p*s < .005.

Thus, the effects of extraversion (but not cognitive style) generalized to participants who did not grasp determinism. In contrast, the effects of cognitive reflection depended entirely on whether participants perceived the universe as deterministic or indeterministic—bolstering our conclusion that cognitive reflection amplifies the effect of perceived sourcehood on free will.
